# Non-operative vs. operative treatment for multiple rib fractures after blunt thoracic trauma: a multicenter prospective cohort study

**DOI:** 10.1007/s00068-022-02093-9

**Published:** 2022-08-25

**Authors:** Ruben J. Hoepelman, Frank. J. P. Beeres, Reinier B. Beks, Arthur A. R. Sweet, Frank F. Ijpma, Koen W. W. Lansink, Bas van Wageningen, Tjarda N. Tromp, Björn-Christian Link, Nicole M. van Veelen, Jochem. M. Hoogendoorn, Mirjam B. de Jong, Mark. C. P. van Baal, Luke P. H. Leenen, Rolf H. H. Groenwold, Roderick M. Houwert

**Affiliations:** 1 Department of Surgery, University Medical Center Utrecht, PO Box 85500, 3508 GA Utrecht, The Netherlands; 2 Department of Orthopedic and Trauma Surgery, Luzerner Kantonsspital, Lucerne, Switzerland; 3 Department of Trauma Surgery, University Medical Center Groningen, University of Groningen, Groningen, The Netherlands; 4 Department of Trauma Surgery, Elisabeth-TweeSteden Hospital, Tilburg, The Netherlands; 5 Department of Trauma Surgery, Radboud University Medical Center, Nijmegen, The Netherlands; 6 Department of Trauma Surgery, Haaglanden Medical Center, The Hague, The Netherlands; 7 Department of Clinical Epidemiology, Leiden University Medical Center, Leiden, The Netherlands; 8 Department of Biomedical Data Sciences, Leiden University Medical Center, Leiden, The Netherlands

**Keywords:** Rib fixation, Multiple rib fractures, Rib fracture, Non-operative treatment

## Abstract

**Background:**

Patients with multiple rib fractures without a clinical flail chest are increasingly being treated with rib fixation; however, high-quality evidence to support this development is lacking.

**Methods:**

We conducted a prospective multicenter observational study comparing rib fixation to non-operative treatment in all patients aged 18 years and older with computed tomography confirmed multiple rib fractures without a clinical flail chest. Three centers performed rib fixation as standard of care. For adequate comparison, the other three centers performed only non-operative treatment. As such clinical equipoise formed the basis for the comparison in this study. Patients were matched using propensity score matching.

**Results:**

In total 927 patients with multiple rib fractures were included. In the three hospitals that performed rib fixation, 80 (14%) out of 591 patients underwent rib fixation. From the nonoperative centers, on average 71 patients were adequately matched to 71 rib fixation patients after propensity score matching. Rib fixation was associated with an increase in hospital length of stay (HLOS) of 4.9 days (95%CI 0.8–9.1, *p* = 0.02) and a decrease in quality of life (QoL) measured by the EQ5D questionnaire at 1 year of 0.1 (95% CI − 0.2–0.0, *p* = 0.035) compared to non-operative treatment. A subgroup analysis of patients who received operative care within 72 h showed a similar decrease in QoL. Up to 22 patients (28%) who underwent surgery experienced implant-related irritation.

**Conclusions:**

We found no benefits and only detrimental effects associated with rib fixation. Based on these results, we do not recommend rib fixation as the standard of care for patients with multiple rib fractures.

**Trial registration:**

Registered in the Netherlands Trial Register NTR6833 on 13/11/2017.

**Supplementary Information:**

The online version contains supplementary material available at 10.1007/s00068-022-02093-9.

## Introduction

Rib fractures are present in 10% of all trauma patients and are, therefore, a very common injury [1]. Despite major advances in trauma and intensive care medicine, it remains a life-threatening injury, with a mortality rate of up to 13% [1]. It is important to distinguish between multiple rib fractures with and without a clinical flail chest, because the former is associated with significant morbidity and increased mortality [1, 2]. Non-operative treatment has long been considered the gold standard for the management of multiple rib fractures. However, in the last decade, there has been growing interest in operative treatments [3, 4]. Rib fixation has been shown to improve short-term outcomes in patients with a clinical flail chest, and as a result, surgeons are broadening the indications for multiple rib fractures without a flail segment, regardless of the lack of evidence [5, 6]. Only one prospective study concerns operative treatment for patients with multiple rib fractures without flail chest; this study failed to show superiority of surgery [7]. Furthermore, the same study showed that randomized controlled trials (RCT) on this subject are not feasible, as 80% of eligible patients declined randomization [7]. This is a commonly encountered problem in (orthopedic) surgical research, and experience has taught us that from design to execution, RCT’s take approximately 10 years to complete, a period in which results may become obsolete [8]. High-quality prospective studies are needed to provide evidence regarding the effects of rib fixation and to determine whether rib fixation should be implemented as a standard of care. Therefore, we conducted this multicenter observational cohort study to compare rib fixation with non-operative treatment in patients with multiple rib fractures without a flail chest.

## Methods

A detailed description of the methods and surgical procedures used in this study is available in the published study protocol [9]. Here, we focus on patients with multiple rib fractures; the results of patients with a flail chest (paradoxical movement of a portion of the chest wall) will be addressed in a separate study. This study adhered to the Strengthening the Reporting of Observational Studies in Epidemiology (STROBE) guidelines [10].

### Study design

This multicenter prospective cohort study included trauma patients admitted to five level 1 trauma centers in the Netherlands and one in Switzerland. All hospitals had roughly similar volumes of trauma patients with comparable injury severities. Annually, approximately 1300 patients, of which 250–400 are polytrauma patients (Injury Severity Score (ISS) > 16), are admitted to the emergency department of each participating center [11]. The study was registered in the Netherlands Trial Registry (NTR6833). Approval from the institutional review board was obtained at every study site. Informed consent was acquired from all participants.

### Patients

Patients aged 18 years or older with computerized tomography (CT) scan confirmed multiple rib fractures (defined as three or more ipsilateral rib fractures) after blunt thoracic trauma were eligible for inclusion. The exclusion criteria were cognitive impairment, non-traumatic rib fractures, and rib fractures due to cardiopulmonary resuscitation.

### Interventions

The patients were treated according to the standard of care of the hospital of admission, which was determined by the attending trauma surgeons. Trauma patients typically receive care from the nearest appropriate hospital according to the protocol in the hospital. As a result, the treatment allocation was, to a large extent, determined by the geographical location of the incident, which was expected to be independent of individual patient characteristics thus creating a natural experiment [12]. Three centers (University Medical Center Utrecht, Luzerner Kantonsspital and Elisabeth-TweeSteden hospital) performed rib fixation as standard of care, according to the algorithm presented in Supplementary Fig. 1. For adequate comparison, the other three centers (University Medical Center Groningen, Radboud University Medical Center and Haaglanden Medical Center) performed only non-operative treatment as standard of care. As such clinical equipoise formed the basis for the comparison in this study. More detailed descriptions of the rib fixation process and postoperative care have been previously described in the published study protocol [9]. Non-operative treatment consisted of adequate pain management, supportive mechanical ventilation when needed, and physiotherapy for breathing exercises, according to standard national guidelines.

### Outcomes

The primary outcome measure was hospital length of stay (HLOS). Secondary outcomes included intensive care unit length of stay (ILOS), duration of mechanical ventilation (DMV), need for tracheostomy, pneumonia rate and other in-hospital complications, in-hospital mortality rate, and general pain (measured using a numeric rating scale [NRS]). Mid- and long-term outcomes were measured at the outpatient clinic visit at 6 weeks and using telephone interviews after 12 months. These measures included pain with breathing and coughing (measured using the NRS), quality of life (measured using the EQ5D-5L), dyspnea burden (measured using the modified Medical Research Council [mMRC] dyspnea scale), and return to work and sports in weeks. Surgery-specific complications included superficial surgical site infection and fracture-related infection (defined by the fracture-related infection [FRI] consensus definitions) [13–15], symptomatic nonunion, and implant removal (assessed using Hulsmans et al.’s algorithm) [16]. Pneumonia was defined as clinical signs and symptoms (two or more present; temperature > 38.5 °C, auscultation with suspicion for infiltrate, thoracic radiographs with signs of infiltrate, leukocytosis, elevated C-reactive protein, or purulent sputum) requiring antimicrobial therapy. Acute respiratory distress syndrome (ARDS) was defined according to the Berlin definition [17]. Superficial surgical site infection was defined as redness, swelling, and/or purulent discharge from the wound. Symptomatic nonunion was defined as the presence of unsuccessfully healed ribs, confirmed by CT scan, at least 6 months after trauma, with clinical evidence of pain. The EQ5D-5L is a standardized instrument for generic health status measurements to assess the quality of life [18]. The mMRC is a five-category scale that characterizes the level of dyspnea with physical activity [19].

### Patient characteristics

Patient characteristics measured at hospital admission included age, sex, body mass index (BMI), American Society of Anesthesia (ASA) score, presence of chronic obstructive pulmonary disease (COPD), smoking status, trauma mechanism, abbreviated injury scale (AIS) score, injury severity score (ISS), number of fractured ribs, severe fracture patterns (defined as three or more sequential rib fractures in two or more places), concomitant injuries (i.e., pulmonary contusion, pneumothorax, hemothorax, sternum, and/or clavicle fracture), and laboratory results (specifically pH and base excess).

### Sample size and statistical analyses

Based on previous studies, the mean HLOS for conservatively treated patients was estimated to be 15 days, with a standard deviation (SD) of 6.3 [20–22]. To detect a difference of 3 days with a power of 80% and a type-I error probability (alpha) of 0.05, accounting for 15% loss after propensity score (PS) matching, 160 patients were needed (i.e., 80 per treatment arm).

All analyses were performed using R statistical software v4.1.2 [23]. Baseline continuous variables are presented as mean with SD or median with interquartile range (IQR). Nominal and categorical data were presented as frequencies and percentages. Differences in the distribution of baseline characteristics between the study groups were quantified using standardized mean differences (SMD). Multiple imputation was applied (25 times) to impute missing values for baseline characteristics: AIS head/face/thorax/extremities (4% [39/927]), base excess (29% [266/927]), BMI (3% [25/927]), ISS (4% [39/927]), pH (29% [265/927]), and smoking status (2% [16/927]), using the ‘mice’ algorithm in R. To control for potential confounding PS matching was performed. The PS was estimated using binary logistic regression analysis, with rib fixation as the dependent variable and age, sex, smoking status, COPD, BMI, ASA score, trauma mechanism, AIS head/face/thorax/abdomen/extremities, ISS, Thorax Trauma Severity Score (TTSS), number of fractured ribs, severe fracture patterns, and concomitant injuries (including pneumothorax, hemothorax, lung contusion, clavicle and sternal fractures) as prespecified covariates in the model. A 1:1 nearest neighbor matching was performed, with a maximum caliper of 0.15 of the SD of the natural logarithm of the PS using the MatchIt algorithm in R. After PS matching, the distributions of the baseline characteristics were compared between the study groups and quantified using SMD. Primary analyses was conducted using a data set of PS-matched subjects. For the primary and secondary outcomes, the relationship between rib fracture fixation and outcomes was assessed using linear regression analysis for continuous outcomes and binary logistic regression analysis for binary outcomes. A sensitivity analysis was performed on the HLOS, which was measured in this analysis from rib fixation to discharge. Follow-up for the non-operative group started 3 days after admission, which is the mean time-to-surgery. This is known as the landmark” method to correct for possible immortal time bias [24]. Subsequently, PS matching and analyses were repeated within a subgroup of patients who received fib fixation within 72 h, as the literature advocates rib fixation within 72 h [25]. In addition to PS matching to correct for confounding, multiple regression analysis was performed, including potential confounders as covariates in the regression model. All patients were included in the analysis, which was a linear or binary logistic regression analysis, depending on the outcome of interest. Analyses were performed separately for each imputed data set and the results were pooled using Rubin’s rules. To check the robustness of multiple imputation, a sensitivity analysis was performed using only complete cases. The statistical analyses plan did not account for multiple testing; hence, the results for secondary outcomes should be interpreted cautiously.

## Results

### Patients

**Fig. 1:**
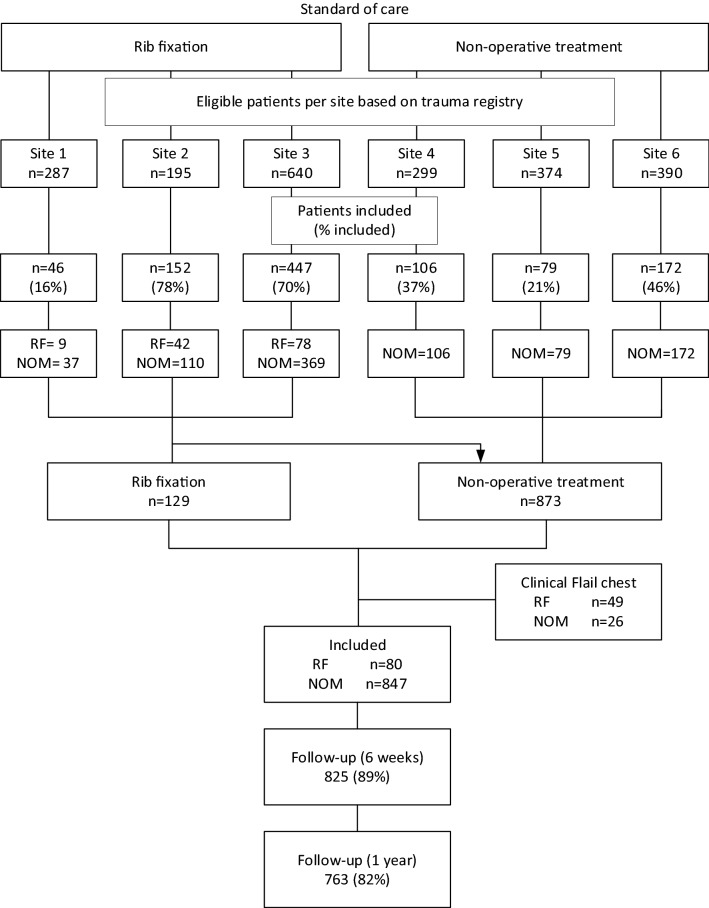
Flow chart of inclusions per study site. *RF* rib fixation, *NOM* nonoperative management

From January 2018 to March 2021, 927 patients with multiple rib fractures were included in all participating centers. In the three hospitals that performed rib fixation, 80 (14%) out of 591 underwent rib fixation. From the nonoperative centers, on average 71 patients were adequately matched after propensity score matching from the imputed data sets. Follow-up was completed in March 2022, with a completion rate of 82% (Fig. 1). Baseline demographic data before and after propensity score matching are available in Table 1, and show adequate matching for all potential confounders. The demographic data and outcomes stratified by treatment site are available in Supplementary Tables 1 and 2. The median duration to rib fixation was 2 days (IQR 1–3.25), the average duration of surgery was 109 min (SD, 79 min), and the average ratio of surgically fixated ribs to the number of rib fractures was 0.45 (Supplementary Table 3). The baseline demographic data of the subgroup analysis were properly matched and are presented in Supplementary Table 4.

**Table 1:** Baseline characteristics before and after propensity score matching

Variable	Before matching				After matching		
	Nonoperative (*n* = 847)	Rib fixation (*n* = 80)	*p* value	SMD	Nonoperative (*n* = 71)*	Rib fixation (*n* = 71)*	SMD**
Age (mean ± SD)	57.6 ± 17.05	63.8 ± 13.4	0.002	0.405	63.5 ± 14.7	63.1 ± 13.6	0.026
Male (*n*, %)	621 (73.3)	64 (80.0)	0.243	0.158	56 (78.5)	55 (77.8)	0.018
ASA-score (*n*, %)			0.257	0.144			0.005
1–2	637 (75.2)	55 (68.8)			48 (67.6)	48 (67.8)	
> 2	210 (24.8)	25 (31.2)			23 (32.4)	23 (32.2)	
BMI (mean ± SD)	26.1 ± 4.5	26.6 ± 4.4	0.352	0.110	26.7 ± 4.5	26.5 ± 4.3)	0.035
COPD (*n*, %)	51 (6.0)	4 (5.0)	0.903	0.045	4 (5.9)	4 (5.6)	0.012
Current smoker (*n*, %)	158 (18.9)	12 (15.6)	0.568	0.089	10.2 (14.3)	11 (15.5)	0.035
Trauma mechanism (*n*, %)			0.429	0.142			0.061
Motor vehicle accident	475 (56.1)	41 (51.2)			34.6 (48.6)	37 (51.6)	
Fall from height/stairs	309 (36.5)	30 (37.5)			28 (40)	26 (37.2)	
Other	63 (7.4)	9 (11.2)			8 (11.4)	8 (11.2)	
ISS (mean ± SD)	18.7 ± 8.9	17.85 ± 7.2	0.429	0.100	18.0 ± 7.3	17.9 ± 7.4	0.007
TTSS (mean ± SD)	8.3 ± 3.0	10.3 ± 3.1	< 0.001	0.655	10.0 ± 3.1	10.1 ± 3.2	0.028
AIS (median, IQR)							
Head	0 (0–2)	0 (0–1)	0.353	0.112	0 (0–1)	0 (0–2)	0.034
Face	0 (0–0)	0 (0–0)	0.592	0.063	0 (0–0)	0 (0–0)	0.012
Thorax	3 (3–3)	3 (3–4)	0.081	0.188	3 (3–3)	3 (3–4)	0.015
Abdomen	0 (0–0)	0 (0–0)	0.024	0.315	0 (0–0)	0 (0–0)	0.002
Extremities	2 (0–2)	2 (0–2)	0.551	0.072	2(0–2)	2 (0–2)	0.008
No. of rib fractures (median, IQR)	6 (4–8)	9 (7–11)	< 0.001	0.921	8 (6–10)	8 (7–10)	0.022
Severe fracture pattern (*n*, %)	133 (15.7)	40 (50.0)	< 0.001	0.756	31 (44.2)	31 (44)	0.005
Bilateral rib fractures (*n*, %)	184 (21.7)	24 (30.0)	0.307	0.132	19 (26.7)	19 (26.5)	0.005
Concomitant thoracic injuries (*n*, %)							
Pulmonary contusion (*n*, %)	318 (37.5)	39 (48.8)	0.065	0.228	33 (46.6)	34 (48.3)	0.034
Pneumothorax (*n*, %)	396 (46.8)	60 (75.0)	< 0.001	0.605	52 (72.8)	51 (71.8)	0.021
Hemothorax (*n*, %)	147 (17.4)	44 (55.0)	< 0.001	0.851	33 (46.6)	35 (49.3)	0.054
Sternum fracture (*n*, %)	82 (9.7)	9 (11.2)	0.799	0.051	8 (11.1)	8 (11.5)	0.012
Clavicle fracture (*n*, %)	164 (19.4)	19 (23.8)	0.426	0.107	15 (20.6)	15 (21)	0.010
Blood pH (mean ± SD)	7.36 ± 0.07	7.35 ± 0.07	0.305	0.141	7.36 ± 0.07	7.36 ± 0.07	0.014
Base excess (mean ± SD)	− 0.63 ± 3.67	− 0.82 ± 3.23	0.693	0.056	− 0.83 ± 3.8	− 0.69 ± 3.4	0.039

*AIS* abbreviated injury score, *ASA-score* American society of anesthesiologists score, *BMI* body mass index, *COPD* chronic obstructive pulmonary disease, *ISS* injury severity score, *MVA* motor vehicle accident, *IQR* interquartile range, *SD* standard deviation, *TTSS* thoracic trauma severity score

*Numbers indicate the average of 25 matched imputed sets, SMD standardized mean difference, **SMD < 0.1 indicates adequate matching

### Primary outcome

After propensity score matching, rib fixation was associated with an increase in HLOS of 4.9 days (95% confidence interval [CI] 0.8–9.1; *p* = 0.019). The median HLOS was 9 days and 12 days for non-operative treatment and rib fixation, respectively (Table 2). Multiple regression analysis of the multiple imputed data sets, while adjusting for confounding factors, yielded similar results (Supplementary Table 5).

**Table 2:** In-hospital outcomes after propensity score matching

Outcome variable	Rib fixation for multiple rib fractures					
Median (IQR) or *n* (%)	Nonoperative*	Rib fixation*	Regression coefficient (*b*)	95% CI	SE	*p* value
Hospital length of stay	9 (6–13)	12 (8–18)	4.9	0.8 to 9.1	2.130	0.019
Hospital length of stay from RF	6 (3–10)	8 (6–15)	4.8	0.8 to 8.9	2.073	0.019
Need for ICU (*n*,%)	23.4 (32.9)	23.4 (32.7)				
ICU length of stay	2 (1–6)	4 (2–11)	1.2	− 0.4 to 2.8	0.823	0.140
Duration of invasive mechanical ventilation	4 (2–5)	6 (3–12.5)	1.0	− 0.1 to 2.1	0.564	0.071
Duration of epidural analgesia	5 (4–6)	4 (3–5)	0.5	− 0.6 to 1.5	0.533	0.382
Duration of intravenous analgesia	3 (1–6)	3 (2–6)	1.0	− 0.7 to 2.6	0.846	0.258
NRS (pain)						
Day 3	3 (2–4)	2 (2–3)	− 0.4	− 1.0 to 0.3	0.334	0.253
Day 5	2 (2–3)	2 (2–4)	0.2	− 0.5 to 0.9	0.357	0.665
Day 7	2 (2–4)	2 (1–4)	0.2	− 0.5 to 0.8	0.339	0.622

Outcome variable	Rib fixation for multiple rib fractures					
In-hospital complications (*n*, %)	Nonoperative*	Rib fixation*	OR	95% CI	SE	*p* value
ARDS	0.4 (0.6)	0 (0.0)	NA	0 to Inf	NA	NA
Tracheostomy	0.5 (0.7)	3 (4.2)	NA	0 to Inf	NA	NA
Pneumonia	12.6 (17.1)	21.4 (30.2)	2.1	0.8 to 5.6	0.494	0.123
Pleural effusion	1.4 (1.9)	3.1 (4.4)	NA	0 to Inf	NA	NA
Pneumothorax	2.6 (3.6)	5.7 (8.1)	NA	0 to Inf	NA	NA
Hemothorax	2.9 (4.1)	5 (7)	1.9	0.3 to 10.4	0.871	0.464
Other complication	20.3 (28.6)	31.4 (43.7)	1.9	0.9 to 4.2	0.397	0.093
Mortality	1.6 (2.3)	1.9 (2.7)	NA	0 to Inf	NA	NA

*RF* rib fixation, *ICU* intensive care unit, *NRS* numeric rating scale, *ARDS* acute respiratory distress syndrome, *IQR* interquartile range, *b* regression coefficient between rib fixation and non-operative treatment, *CI* confidence interval, *OR* odd ratio, *SE* standard deviation, *NA* no answer (due to small numbers)

*Numbers indicate the average of 25 matched imputed sets

### Secondary outcomes

Rib fixation was associated with a decrease in EQ5D-5L index value at 1 year follow-up of 0.1 (95% CI − 0.2–0.0, *p* = 0.035). No relationship was observed between rib fixation and in-hospital, mid-, or long-term complications, or with other secondary outcomes. The overall mortality was low and did not differ between the treatments (2.3% vs. 2.7%). Patients appeared to develop pneumonia more frequently after rib fixation (30.2% vs. 17.1%), although this difference was not statistically significant (*p* = 0.123). The rates of other (pulmonary) complications and mortality were low in both treatment groups. All outcomes are presented in Tables 2 and 3. Twenty-two patients (28%) experienced implant-related irritation after rib fixation. Supplementary Table 6 shows all outcomes before propensity score matching.

**Table 3:** Mid- and long-term outcomes after propensity score matching

Mid- and long-term outcomes	Rib fixation for multiple rib fractures					
	Nonoperative*	Rib fixation*	Regression coefficient (*b*)	95% CI	SE	*p* value
*Follow-up 6 weeks*						
EQ5D-5L index value, mean ± SD	0.72 ± 0.2	0.71 ± 0.2	− 0.0	− 0.1 to 0.1	0.038	0.778
EQ5D-5L VAS, mean ± SD	66.2 ± 17	67.3 ± 17	1.1	− 5.8 to 8.1	3.546	0.750
MMRC, median (IQR)	0 (0–1)	0 (0–1)	0.2	− 0.3 to 0.6	0.231	0.437
NRS						
General	2 (1–3)	2 (1–4)	0.3	− 0.5 to 1.1	0.414	0.416
Breathing	0 (0–2)	1 (0–3)	0.7	− 0.1 to 1.5	0.393	0.063
Coughing	2 (0–4)	3 (1–5)	0.7	− 0.3 to 1.8	0.520	0.155

Complications (*n*, %)*	Rib fixation for multiple rib fractures					
	Nonoperative*	Rib fixation*	OR	95% CI	SE	*p* value
Pneumonia	0.2 (0.3)	0.1 (0.2)	NA	NA	NA	NA
Pleural effusion	1.3 (1.8)	1 (1.4)	NA	NA	NA	NA
Pneumothorax	0.1 (0.1)	0 (0)	NA	NA	NA	NA
Hemothorax	0 (0)	0 (0)	NA	NA	NA	NA
*Follow-up 1 year*						
EQ5D-5L index value, mean ± SD	0.81 ± 0.2	0.74 ± 0.2	− 0.1	− 0.2 to 0.0	0.042	0.035
EQ5D-5L VAS, mean ± SD	74.8 ± 19	72.4 ± 17	− 3.0	− 11.1 to 5.1	4.119	0.463
MMRC, median (IQR)	0 (0–1)	0 (0–1)	0.2	− 0.2 to 0.5	0.163	0.334
NRS (pain)						
General	0 (0–2)	2 (0–4)	0.9	− 0.1 to 1.8	0.469	0.064
Breathing	0 (0–0)	0 (0–0)	0.1	− 0.3 to 0.4	0.175	0.704
Coughing	0 (0–0)	0 (0–1)	0.8	0.1 to 1.5	0.342	0.054

Complications	Rib fixation for multiple rib fractures					
	Nonoperative*	Rib fixation*	OR	95% CI	SE	*p* value
Implant related irritation (*n*,%)	0 (0)	19 (27)	NA	NA	NA	NA
Implant removal (*n*, %)	0 (0)	1.6 (2.2)	NA	NA	NA	NA
Symptomatic non-union (*n*, %)	1.2 (1.8)	1 (1.4)	NA	NA	NA	NA
Deceased (*n*, %)	1.3 (1.9)	0.8 (1.1)	NA	NA	NA	NA
Return to work (weeks), median (IQR)	12 (7–20)	12 (10–20)	0.1	− 3.7 to 3.6	1.873	0.969
Return to sports (weeks), median (IQR)	14 (8–26)	12 (10–20)	− 0.7	− 4.5 to 3.0	1.917	0.704

*IQR* interquartile range, *b* regression coefficient between rib fixation and non-operative treatment, *CI* confidence interval, *SE* standard deviation, *NA* no answer (due to small numbers), *MMRC* modified medical research council dyspnea scale, *NR*S numeric rating scale

*Data shown is the average of 25 matched imputed sets

### Subgroup and sensitivity analyses

A propensity score matched analysis of patients who received rib fixation within 72 h or were conservatively treated revealed no association between rib fixation and HLOS (3.5 days, 95% CI − 1.6–8.6, *p* = 0.18). Rib fixation was associated with a decrease in the EQ5D-5L index score at 1 year follow-up by 0.09 (95% CI − 0.2 to − 0.0, *p* = 0.043). No relationship was observed between rib fixation and any other secondary outcome (Table 4). The pneumonia rates were 26.4% and 17.1% in the rib fixation and non-operative groups, respectively (*p* = 0.313). The outcomes of subgroup analysis are presented in Table 4.

**Table 4:** Outcomes after propensity score matching with rib fixation within 72 h

Outcome variable	Rib fixation for multiple rib fractures					
Median (IQR) or *n* (%)	Nonoperative*	Rib fixation*	Regression coefficient (*b*)	95% CI	SE	*p* value
Hospital length of stay	9 (6–13)	10 (8–16)	3.5	− 1.6 to 8.6	2.597	0.175
Hospital length of stay from RF	6 (3–10)	8 (6–16)	5.0	− 0.1 to 10.1	2.599	0.052
Need for ICU (*n*,%)	17.7 (33.5)	14.6 (27.6)				
ICU length of stay	2 (1–6)	4 (2–14)	0.7	− 1.0 to 2.5	0.898	0.427
Need for ventilation (*n*,%)	8.4 (15.8)	11.6 (22)				
Duration of Invasive mechanical ventilation	3 (1–5)	6 (3–14)	1.1	− 0.2 to 2.4	0.657	0.096
Epidural treatment (*n*, %)	14.3 (27.1)	12.0 (22.6)				
Duration of epidural analgesia	5 (4–6)	3 (2–4.5)	− 0.5	− 1.7 to 0.8	0.651	0.474
Duration of Intravenous analgesia	3 (1–6)	3 (2–5)	0.5	− 1.2 to 2.3	0.875	0.537
*NRS (pain)*						
Day 3	3 (1–6)	3 (2–5)	− 0.3	− 1.1 to 0.5	0.416	0.494
Day 5	3 (2–4)	3 (2–4)	0.1	− 0.9 to 0.7	0.423	0.831
Day 7	2 (2–3)	2 (1–4)	0.1	− 0.7 to 0.9	0.407	0.836

Outcome variable	Rib fixation for multiple rib fractures					
In-hospital complications (*n*, %)	Nonoperative*	Rib fixation*	OR	95% CI	SE	*p* value
ARDS	0.6 (1.1)	0 (0.0)	NA	0 to Inf	NA	NA
Tracheostomy	0.6 (1.2)	3 (5.7)	NA	0 to Inf	NA	NA
Pneumonia	9.0 (17.1)	14.0 (26.4)	1.8	0.6 to 5.5	0.573	0.313
Pleural effusion	1.3 (2.5)	2 (3.8)	NA	0 to Inf	NA	NA
Pneumothorax	1.6 (3)	2 (3.8)	NA	0 to Inf	NA	NA
Hemothorax	2.4 (4.5)	4 (7.6)	NA	0 to Inf	NA	NA
Other complication	13.5 (25.6)	19.9 (37.7)	1.8	0.7 to 4.8	0.501	0.248
Mortality	1.4 (2.6)	1.9 (3.6)	NA	0 to Inf	NA	NA
*Follow-up 1 year*						
EQ5D-5L index value, mean ± SD	0.81 ± 0.2	0.73 ± 0.2	− 0.09	− 0.2 to 0.0	0.049	0.043
EQ5D-5L VAS, mean ± SD	74.1 ± 19	72.6 ± 16	− 3.7	− 12.6 to 5.1	3.806	0.454
MMRC, median (IQR)	0 (0–1)	0 (0–1)	0.1	− 0.2 to 0.5	0.185	0.409

*RF* rib fixation, *ICU* intensive care unit, *IMV* invasive mechanical ventilation, *IV* intravenous, *NRS* numeric rating scale, *ARDS* acute respiratory distress syndrome, *IQR* interquartile range, *b* regression coefficient between rib fixation and non-operative treatment, *CI* confidence interval, *SE* standard deviation, *NA* no answer (due to small numbers)

*Numbers indicate the average of 25 matched imputed sets

The complete case analysis yielded similar results with regard to hospital length of stay as the primary analyses (Supplementary Table 5).

## Discussion

Rib fixation for multiple rib fractures failed to show any benefit over non-operative treatment. Rib fixation prolonged the hospital stay by 4.9 days (95% CI 0.8–9.1), was associated with a decrease in quality of life after 1 year (EQ5D-5L score − 0.1, 95% CI − 0.2–0.0), and 28% of the patients experienced implant-related irritation.

This is the largest study to date with 927 prospectively enrolled patients, which addresses this research question. Decreasing HLOS is a fundamental issue because of pressure on healthcare in general. The HLOS reflects the results of different medical problems in the heterogeneous group of patients with multiple rib fractures. As no association was found between ILOS, DMV, and mortality, the prolonged HLOS can safely be attributed to rib fixation instead of environmental factors. It is possible that HLOS was increased in patients who underwent rib fixation, because they were hospitalized several days before surgery; As was hypothesized by many previous studies [22, 26, 27]. However, when adjusted for time-to-operation, outcomes were similar which makes this hypothesis less likely. Another explanation could be that the anesthesia inherent to and physiological stress induced by surgery inhibit recovery time compared to nonoperative patients [28]. Previous studies have reported contrasting results regarding HLOS. Two studies found roughly similar durations of HLOS as our study (10 days vs. 12 days, and 6.5 days vs. 12.7 days, both in favor of non-operative treatment) [29, 30]. However, the first study did not find a statistically significant association [29], which might be attributable to the small sample size, whereas the second study emphasized the possibility of residual confounding [30]. One meta-analysis reported a reduced mean difference of 5.8 days after rib fixation [6]. The results of this meta-analysis should be interpreted cautiously, because out of the nine studies included in this meta-analysis, only four were used to estimate effects regarding this outcome, there was significant heterogeneity between the studies, and none of the studies corrected for confounding.

A decrease in quality of life after rib fixation was the second association we found. No other study has investigated the quality of life 1 year after rib fixation for multiple rib fractures. The average EQ5D-5L score for the Dutch population was 0.839 for people aged ≥ 60 years [31]. Studies have suggested that trauma patients are not completely representative of the general population [32]; however, while the non-operative group was close to this average (0.82, SD 0.2), the rib fixation group was not (0.74, SD 0.2). We hypothesized that the high rate of implant irritation (28%) was of influence on the decrease in QoL. A subgroup analysis comparing QoL in patients with and without implant irritation strengthens this hypothesis (0.69 ± 0.2 vs. 0.77 ± 0.2); however, this was an underpowered subgroup analysis. Furthermore, many of these patients were considering implant removal. Since our follow-up was limited to 1 year, we could not study the influence of implant removal. Future studies are needed to determine whether there indeed is an association.

Regarding ILOS, DMV, pneumonia rate, and mortality, no statistically significant association with rib fixation was found, which is consistent with recently published prospective studies [7, 33]. It is worth noting that the pneumonia rate was considerably higher than in other studies, which is most likely explained by the varying definitions used for pneumonia in clinical research. Furthermore, the differences between rib fixation and non-operative treatment for pneumonia rate (30.1% vs. 17.1%) and ILOS (4 days vs. 2 days) were considerable, even though they were not statistically significant. The sample size was not adjusted for these outcomes, and may, therefore, be insufficient. The mortality rate, however, was low, similar to that in recent national registry studies [1, 2]. Mortality rates after rib fractures have declined over the past decade, most likely due to the optimization of acute trauma and intensive care management [34, 35].

Therefore, these studies have shifted their focus to other outcomes. One study analyzed pain at 2 weeks follow-up and found a statistically significant difference in 1.5 NRS in favor of rib fixation; however, there was no reduction in narcotic use [7]. Although pain and potential consequential pneumonia are important factors in the treatment of rib fractures, this outcome alone is not sufficient to justify rib fixation, as the benefits should clearly outweigh the costs and risks before it should be considered.

This study had several potential limitations. Although we were able to correct for many confounders, the possibility of unmeasured confounding exists. However, we believe we have included the most relevant potential confounders in our analyses, and therefore, the potential impact of unmeasured confounding is expected to be limited. For all observed confounders, the SMD was below 0.1 after matching, indicating that the measured confounding was adequately controlled for [36]. Moreover, multiple regression analysis showed similar results, thus providing evidence of the robustness of results against different modelling assumptions.

A second limitation that must be mentioned, are the indications for rib fixation. Thoracic deformity and inadequate pain management were always assessed by the attending trauma surgeon(s); however, they were clinical assessments and, therefore, were subjective to some extent. It does, however, resemble daily clinical practice.

Our study shows the feasibility of observational studies in trauma surgery. Alternatively, we could have performed a randomized controlled trial, where we would not have the potential limitation of unmeasured confounding. However, the recent study on rib fractures by Pieracci et al. illustrates the challenges that may occur in randomized studies in trauma surgery 80% of the eligible subjects declined randomization [7]. The feasibility of large orthopedic trials has been questioned previously [37]. Observational studies often do not suffer from these hardships and may better represent daily clinical practice [38]. Our study is exemplarily of the potential of observational studies, while 4 years were projected, the inclusion was finished in three, and has resulted in the largest prospective cohort of patients with multiple rib fractures to date. One aspect worth mentioning here is the large variation in inclusion rates between participating centers, which ranged from 16 to 78%, which supports the notion that the inclusion rate in observational trauma surgery studies is still subject to “enthusiasm” of the investigators as immaculately worded by Jeray et al. [39].

In conclusion, we found no benefit of rib fixation over non-operative treatment. Based on these results, we do not recommend rib fixation as the standard of care for patients with multiple rib fractures.

## Supplementary Information

Below is the link to the electronic supplementary material.

Supplementary file1 (DOCX 128 KB)

Supplementary file2 (DOCX 26 KB)

Supplementary file3 (DOCX 26 KB)

Supplementary file4 (DOCX 16 KB)

Supplementary file5 (DOCX 21 KB)

Supplementary file6 (DOCX 22 KB)

Supplementary file7 (DOCX 21 KB)
