# Supplementary material for: Non-operative vs. operative treatment for multiple rib fractures after blunt thoracic trauma: a multicenter prospective cohort study
Source: Eur J Trauma Emerg Surg. 2022 Aug 25;49(1):461–71. doi: 10.1007/s00068-022-02093-9 (PMC9925506; doi:10.1007/s00068-022-02093-9)
Supplement: Supplementary file 1 — Supplementary file1 (DOCX 128 KB) [file 68_2022_2093_MOESM1_ESM.docx]

**Supplementary Figure 1.** Clinical based algorithm for the treatment of multiple rib fractures.


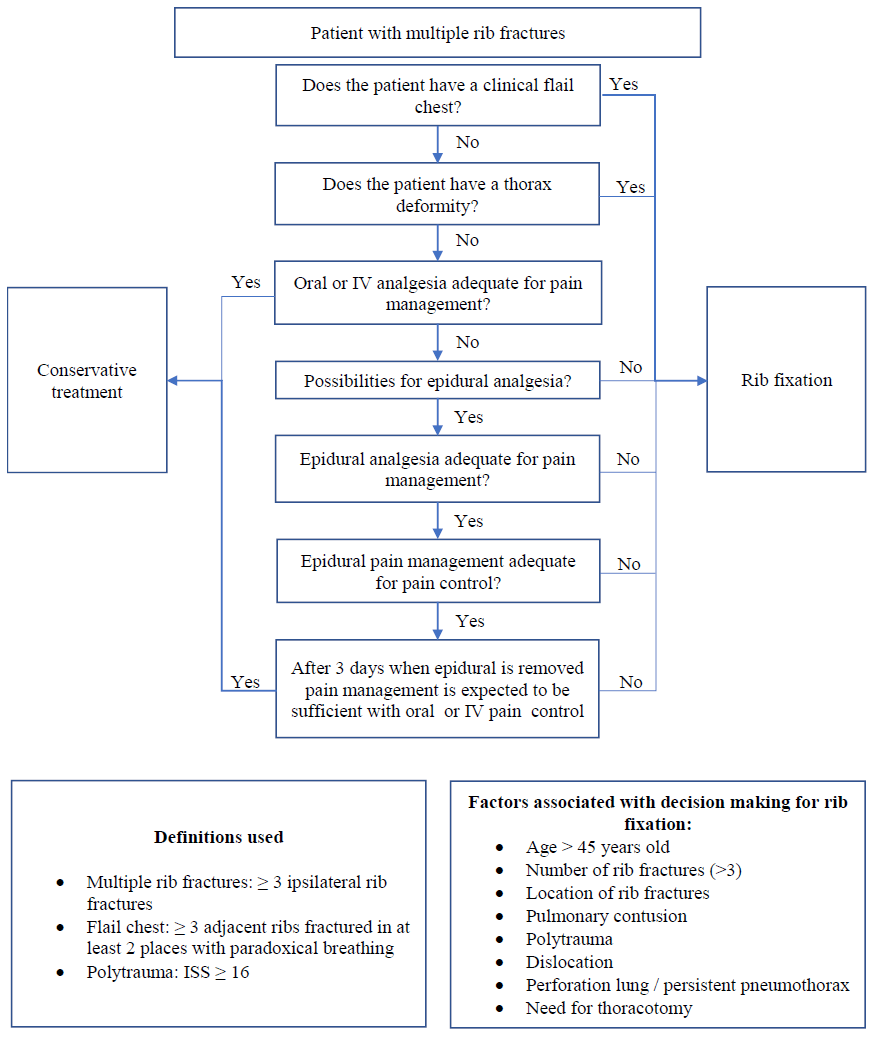


ISS injury severity score; IV intravenous.

**Non-operative versus operative treatment for multiple rib fractures after blunt thoracic trauma: a multicenter prospective cohort study**

European Journal of Trauma and Emergency Surgery

Ruben J. Hoepelman,^1^ MD, Frank. J.P. Beeres,^2^ MD, PD, PhD, FEBS, Reinier B. Beks,^1^ MD, PhD, Arthur A.R. Sweet,^1^ MD, Frank F. Ijpma,^3^ MD, PhD, FEBS, Koen W.W. Lansink^4^, MD, PhD, Bas van Wageningen,^5^ MD, Tjarda N. Tromp,^5^ Bsc, Björn-Christian Link,^2^ MD, PhD, Nicole M. van Veelen,^2^ MD, Jochem. M. Hoogendoorn,^6^ MD, PhD Mirjam B. de Jong,^1^ MD, PhD, Mark. C.P. van Baal,^1^ MD, PhD , Luke P.H Leenen,^1^ MD, PhD, FACS, FEBS, Rolf H.H. Groenwold,^7,8^ MD, PhD, and Roderick M. Houwert,^1^ MD, PhD

1. Department of Trauma Surgery, University Medical Center Utrecht, Utrecht, the Netherlands
2. Department of Orthopedic and Trauma Surgery, Luzerner Kantonsspital, Lucerne, Switzerland
3. Department of Trauma surgery, University Medical Center Groningen, University of Groningen, Groningen, the Netherlands
4. Department of Trauma Surgery, Elisabeth-TweeSteden hospital, Tilburg, The Netherlands.
5. Department of Trauma Surgery, Radboud University Medical Center, Nijmegen, the Netherlands
6. Department of Trauma Surgery, Haaglanden Medical Center, the Hague, the Netherlands
7. Department of Clinical Epidemiology, Leiden University Medical Center, Leiden, the Netherlands
8. Department of Biomedical Data Sciences, Leiden University Medical Center, Leiden, the Netherlands

**Corresponding author**

Roderick M. Houwert, MD, PhD

E-mail address: r.m.houwert@umcutrecht.nl
