# Supplementary material for: Non-operative vs. operative treatment for multiple rib fractures after blunt thoracic trauma: a multicenter prospective cohort study
Source: Eur J Trauma Emerg Surg. 2022 Aug 25;49(1):461–71. doi: 10.1007/s00068-022-02093-9 (PMC9925506; doi:10.1007/s00068-022-02093-9)
Supplement: Supplementary file 3 — Supplementary file3 (DOCX 26 KB) [file 68_2022_2093_MOESM3_ESM.docx]

**Supplementary Table 2.** In-hospital outcomes per treatment site

| Outcomes  (Median (IQR) or mean ±SD) | Site 1 | |  | Site 2 | |  | Site 3 | |  | Site 4 |  | Site 5 |  | Site 6 |
| --- | --- | --- | --- | --- | --- | --- | --- | --- | --- | --- | --- | --- | --- | --- |
|  | Non operative | Rib fixation |  | Non operative | Rib fixation |  | Non operative | Rib fixation |  | Non Operative |  | Non Operative |  | Non Operative |
| Number of patients | 37 | 4 |  | 108 | 22 |  | 366 | 54 |  | 106 |  | 77 |  | 153 |
| Hospital length of stay | 6 (4-10) | 15 (8-27) |  | 8 (4-13) | 12 (8-14) |  | 8 (4-13) | 13 (8-18) |  | 4 (3-11) |  | 5 (4-9) |  | 9 (6-19) |
| Hospital length of stay from RF | 6 (4-10) | 12 (7-18) |  | 8 (4-13) | 6 (4-9) |  | 8 (4-13) | 11 (7-17) |  | 4 (3-11) |  | 5 (4-9) |  | 9 (6-19) |
| ICU treatment (n, %) | 9 (24) | 4 (100) |  | 38 (35) | 9 (41) |  | 74 (20) | 14 (26) |  | 39 (36) |  | 17 (22) |  | 51 (33) |
| ICU length of stay | 1 (1-3) | 9 (2-20) |  | 2 (2-4) | 4 (2-7) |  | 3 (1-6) | 6.5 (4-13) |  | 1 (1-3) |  | 2 (1-6) |  | 4 (1-6) |
| Need for ventilation (n, %) | 1 (3) | 3 (75) |  | 6 (6) | 4 (18) |  | 63 (17) | 13 (24) |  | 5 (5) |  | 9 (12) |  | 31 (20) |
| Duration of IMV | 1 (1-1) | 13 (8-14) |  | 1 (1-1) | 2 (2-2) |  | 2 (1-6) | 6 (3-11) |  | 2 (1-5) |  | 2 (1-5) |  | 3 (2-5) |
| Tracheotomy | 0 (0-0) | 0 (0-0) |  | 0 (0-0) | 0 (0) |  | 8 (2) | 3 (6) |  | 0 (0) |  | 1 (1) |  | 0 (0) |
| Epidural treatment (n, %) | 18 (49) | 3 (75) |  | 29 (27) | 17 (77) |  | 20 (6) | 6 (11) |  | 8 (8) |  | 22 (29) |  | 42 (28) |
| Duration of epidural analgesia | 4 (3-5) | 8 (6-9) |  | 6 (6-9) | 5 (4-10) |  | 4 (3-5) | 2.5 (2-4) |  | 4 (2-5) |  | 5 (4-6) |  | 5 (4-7) |
| Duration of intravenous analgesia | 1 (0-4) | 7 (3-11) |  | 1 (1-3) | 2 (1-4) |  | 2 (0-4) | 4 (3-7) |  | 1 (0-3) |  | 2 (1-4) |  | 5 (3-8) |
| NRS (pain) day 3 | 3 (2-4) | 3 (2-4) |  | 1 (0-3) | 1 (0-2) |  | 2 (1-3) | 2 (2-4) |  | 1 (0-2) |  | 2 (2-4) |  | 2 (1-3) |
| NRS (pain) day 5 | 5 (3-5) | 4 (4-5) |  | 1 (0-2) | 1 (0-2) |  | 2 (1-4) | 2 (1-4) |  | 1 (0-2) |  | 2 (1-3) |  | 2 (1-3) |
| NRS (pain) day 7 | 1 (0-1) | 2 (1-2) |  | 1 (0-2) | 0 (0-1) |  | 2 (1-4) | 2 (1-4) |  | 1 (0-2) |  | 3 (2-4) |  | 2 (1-3) |
| Days to Surgery, median (IQR) | - | 3 (1-8) |  | - | 3.5 (2-6) |  | - | 1 (1-3) |  | - |  | - |  | - |
| Duration of Surgery (minutes), mean ±SD | - | 72 ±86 |  | - | 188 ±101 |  | - | 90 ±41 |  | - |  | - |  | - |
| Rib fixation < 72 hours (n, %) | - | 2 (50) |  | - | 11 (50) |  | - | 47 (87) |  | - |  | - |  | - |
| Rib fixation <48 hours (n, %) | - | 2 (50) |  | - | 7 (32) |  | - | 40 (74) |  | - |  | - |  | - |
| Rib fixation indication (n, %) |  |  |  |  |  |  |  |  |  |  |  |  |  |  |
| Pain | - | 2 (50) |  | - | 2 (9) |  | - | 34 (63) |  | - |  | - |  | - |
| Thorax deformity | - | 1 (25) |  | - | 10 (46) |  | - | 8 (15) |  | - |  | - |  | - |
| Perforated Lung/pneumothorax | - | 0 (0) |  | - | 4 (18) |  | - | 1 (2) |  | - |  | - |  | - |
| Thoracotomy | - | 0 (0) |  | - | 0 (0) |  | - | 5 (9) |  | - |  | - |  | - |
| Other | - | 1 (25) |  | - | 6 (27) |  | - | 6 (11) |  | - |  |  |  | - |
| Complications (n, %) |  |  |  |  |  |  |  |  |  |  |  |  |  |  |
| ARDS | 0 (0) | 0 (0) |  | 0 (0) | 0 (0) |  | 0 (0) | 0 (0) |  | 1 (1) |  | 1 (1) |  | 0 (0) |
| Pneumonia | 5 (14) | 2 (50) |  | 8 (7) | 3 (14) |  | 54 (15) | 19 (35) |  | 8 (8) |  | 11 (14) |  | 27 (18) |
| Pneumothorax | 0 (0) | 0 (0) |  | 4 (4) | 2 (9) |  | 7 (2) | 4 (7) |  | 4 (4) |  | 1 (1) |  | 2 (1) |
| Pleural effusion | 0 (0) | 0 (0) |  | 5 (5) | 2 (9) |  | 4 (1) | 2 (4) |  | 0 (0) |  | 4 (5) |  | 5 (3) |
| Hemothorax | 0 (0) | 0 (0) |  | 3 (3) | 1 (5) |  | 10 (3) | 4 (7) |  | 1 (1) |  | 1 (1) |  | 1 (1) |
| Empyema | 0 (0) | 0 (0) |  | 0 (0) | 0 (0) |  | 0 (0) | 0 (0) |  | 1 (1) |  | 0 (0) |  | 1 (1) |
| Tension pneumothorax | 0 (0) | 0 (0) |  | 0 (0) | 0 (0) |  | 2 (1) | 1 (2) |  | 0 (0) |  | 0 (0) |  | 0 (0) |
| Fracture related infection | 0 (0) | 0 (0) |  | 0 (0) | 0 (0) |  | 0 (0) | 0 (0) |  | 0 (0) |  | 0 (0) |  | 0 (0) |
| Surgical site infection | 0 (0) | 0 (0) |  | 0 (0) | 0 (0) |  | 0 (0) | 0 (0) |  | 0 (0) |  | 0 (0) |  | 0 (0) |
| Other complication (n, %) | 5 (14) | 2 (50) |  | 10 (9) | 5 (23) |  | 108 (30) | 29 (54) |  | 6 (6) |  | 21 (27) |  | 38 (25) |
| Mortality (n, %) | 0 (0) | 0 (0) |  | 0 (0) | 0 (0) |  | 7 (2) | 2 (4) |  | 1 (1) |  | 1 (1) |  | 4 (3) |

RF rib fixation, ICU intensive care unit, IMV invasive mechanical ventilation, IV intravenous, NRS numeric rating scale, ARDS, acute respiratory distress syndrome, IQR interquartile range

**Non-operative versus operative treatment for multiple rib fractures after blunt thoracic trauma: a multicenter prospective cohort study**

European Journal of Trauma and Emergency Surgery

Ruben J. Hoepelman,^1^ MD, Frank. J.P. Beeres,^2^ MD, PD, PhD, FEBS, Reinier B. Beks,^1^ MD, PhD, Arthur A.R. Sweet,^1^ MD, Frank F. Ijpma,^3^ MD, PhD, FEBS, Koen W.W. Lansink^4^, MD, PhD, Bas van Wageningen,^5^ MD, Tjarda N. Tromp,^5^ Bsc, Björn-Christian Link,^2^ MD, PhD, Nicole M. van Veelen,^2^ MD, Jochem. M. Hoogendoorn,^6^ MD, PhD Mirjam B. de Jong,^1^ MD, PhD, Mark. C.P. van Baal,^1^ MD, PhD , Luke P.H Leenen,^1^ MD, PhD, FACS, FEBS, Rolf H.H. Groenwold,^7,8^ MD, PhD, and Roderick M. Houwert,^1^ MD, PhD

1. Department of Trauma Surgery, University Medical Center Utrecht, Utrecht, the Netherlands
2. Department of Orthopedic and Trauma Surgery, Luzerner Kantonsspital, Lucerne, Switzerland
3. Department of Trauma surgery, University Medical Center Groningen, University of Groningen, Groningen, the Netherlands
4. Department of Trauma Surgery, Elisabeth-TweeSteden hospital, Tilburg, The Netherlands.
5. Department of Trauma Surgery, Radboud University Medical Center, Nijmegen, the Netherlands
6. Department of Trauma Surgery, Haaglanden Medical Center, the Hague, the Netherlands
7. Department of Clinical Epidemiology, Leiden University Medical Center, Leiden, the Netherlands
8. Department of Biomedical Data Sciences, Leiden University Medical Center, Leiden, the Netherlands

**Corresponding author**

Roderick M. Houwert, MD, PhD

E-mail address: r.m.houwert@umcutrecht.nl
