# Supplementary material for: Non-operative vs. operative treatment for multiple rib fractures after blunt thoracic trauma: a multicenter prospective cohort study
Source: Eur J Trauma Emerg Surg. 2022 Aug 25;49(1):461–71. doi: 10.1007/s00068-022-02093-9 (PMC9925506; doi:10.1007/s00068-022-02093-9)
Supplement: Supplementary file 5 — Supplementary file5 (DOCX 21 KB) [file 68_2022_2093_MOESM5_ESM.docx]

**Supplementary Table 4** Baseline characteristics after propensity score matching with rib fixation within 72 hours

| Variable | Nonoperative (n=53)* | Rib fixation (n=53)* | SMD** |
| --- | --- | --- | --- |
| Age (mean ±SD) | 64.1 ±14 | 63.5 ±13.8 | 0.044 |
| Male (n, %) | 41 (77.6) | 40 (75.6) | 0.047 |
| ASA-score (n, %) |  |  | 0.037 |
| 1-2 | 37 (70) | 38 (71.7) |  |
| >2 | 16 (30) | 15 (28.3) |  |
| BMI | 27.2 ±4.6 | 26.9 ±4.5 | 0.081 |
| COPD (n, %) | 2 (3.9) | 2 (4.1) | 0.008 |
| Current smoker (n, %) | 7 (14) | 9 (17.1) | 0.086 |
| Trauma mechanism (n, %) |  |  | 0.083 |
| MVA | 29 (54.1) | 29 (55.2) |  |
| Fall | 18 (34.7) | 17 (31.6) |  |
| Other | 6 (11.2) | 7 (13.2) |  |
| ISS (mean ±SD) | 18.0 ±7.3 | 18.2 ±8.0 | 0.027 |
| TTSS (mean ±SD) | 10.2 ±3.0 | 10.3 ±3.2 | 0.035 |
| AIS (median, IQR) |  |  |  |
| Head | 0 (0-2) | 0 (0-1) | 0.013 |
| Face | 0 (0-0) | 0 (0-0) | 0.046 |
| Thorax | 3 (3-3) | 3 (3-3) | 0.017 |
| Abdomen | 0 (0-0) | 0 (0-0) | 0.039 |
| Extremities | 2 (0-2) | 2 (1-2) | 0.066 |
| No. of rib fractures (median, IQR) | 8 (7-10) | 8 (7-11) | 0.036 |
| Severe fracture pattern (n, %) | 25 (47.4) | 24 (45.5) | 0.038 |
| Bilateral rib fractures (n, %) | 12 (23.5) | 14 (26.9) | 0.077 |
| Concomitant (thoracic) injuries (n, %) |  |  |  |
| Pulmonary contusion (n, %) | 24 (47.4) | 27 (51.5) | 0.082 |
| Pneumothorax (n, %) | 37 (69.7) | 38 (71.6) | 0.042 |
| Hemothorax (n, %) | 23 (43.3) | 23 (43.3) | <0.001 |
| Sternum fracture (n, %) | 7 (13) | 6 (12.3) | 0.023 |
| Clavicle fracture (n, %) | 11 (20.1) | 11 (20.5) | 0.009 |
| Blood pH (mean ±SD) | 7.36 ±0.06 | 7.36 ±0.07 | 0.030 |
| Base excess (mean ±SD) | -0.87 ±3.6 | -0.87 ±3.1 | 0.002 |

*Numbers indicate the average of 25 matched imputed sets, SMD standardized mean difference, **SMD <0.1 indicates adequate matching, ASA American society of anesthesiologists, Chronic Obstructive Pulmonary Disease, ISS injury severity score, MVA motor vehicle accident, AIS abbreviated injury score, IQR interquartile range, SD standard deviation, TTSS thoracic trauma severity score

**Non-operative versus operative treatment for multiple rib fractures after blunt thoracic trauma: a multicenter prospective cohort study**

European Journal of Trauma and Emergency Surgery

Ruben J. Hoepelman,^1^ MD, Frank. J.P. Beeres,^2^ MD, PD, PhD, FEBS, Reinier B. Beks,^1^ MD, PhD, Arthur A.R. Sweet,^1^ MD, Frank F. Ijpma,^3^ MD, PhD, FEBS, Koen W.W. Lansink^4^, MD, PhD, Bas van Wageningen,^5^ MD, Tjarda N. Tromp,^5^ Bsc, Björn-Christian Link,^2^ MD, PhD, Nicole M. van Veelen,^2^ MD, Jochem. M. Hoogendoorn,^6^ MD, PhD Mirjam B. de Jong,^1^ MD, PhD, Mark. C.P. van Baal,^1^ MD, PhD , Luke P.H Leenen,^1^ MD, PhD, FACS, FEBS, Rolf H.H. Groenwold,^7,8^ MD, PhD, and Roderick M. Houwert,^1^ MD, PhD

1. Department of Trauma Surgery, University Medical Center Utrecht, Utrecht, the Netherlands
2. Department of Orthopedic and Trauma Surgery, Luzerner Kantonsspital, Lucerne, Switzerland
3. Department of Trauma surgery, University Medical Center Groningen, University of Groningen, Groningen, the Netherlands
4. Department of Trauma Surgery, Elisabeth-TweeSteden hospital, Tilburg, The Netherlands.
5. Department of Trauma Surgery, Radboud University Medical Center, Nijmegen, the Netherlands
6. Department of Trauma Surgery, Haaglanden Medical Center, the Hague, the Netherlands
7. Department of Clinical Epidemiology, Leiden University Medical Center, Leiden, the Netherlands
8. Department of Biomedical Data Sciences, Leiden University Medical Center, Leiden, the Netherlands

**Corresponding author**

Roderick M. Houwert, MD, PhD

E-mail address: r.m.houwert@umcutrecht.nl
