# Supplementary material for: Non-operative vs. operative treatment for multiple rib fractures after blunt thoracic trauma: a multicenter prospective cohort study
Source: Eur J Trauma Emerg Surg. 2022 Aug 25;49(1):461–71. doi: 10.1007/s00068-022-02093-9 (PMC9925506; doi:10.1007/s00068-022-02093-9)
Supplement: Supplementary file 6 — Supplementary file6 (DOCX 22 KB) [file 68_2022_2093_MOESM6_ESM.docx]

**Supplementary Table 5.** Outcomes analyzed with multiple linear regression model with covariates

| Outcome variable |  |  | Complete case analysis |  |  |  |  | Multiple imputed data |  |  |  |
| --- | --- | --- | --- | --- | --- | --- | --- | --- | --- | --- | --- |
|  | Nonoperative (n=847) | Rib fixation (n=80) | Regression coefficient (b) | 95% CI | SE | *p* value |  | regression coefficient (b) | 95% CI | SE | *p* value |
| Hospital length of stay | 7 (4-13) | 12 (8-18) | 4.4 | 1.4 to 7.5 | 1.5 | 0.004 |  | 4.5 | 1.9 to 7.0 | 1.3 | <0.001 |
| Hospital length of stay from RF | 6 (2-12) | 8 (6-15) | 4.6 | 1.6 to 7.6 | 1.5 | 0.003 |  | 4.3 | 1.8 to 6.8 | 1.3 | <0.001 |
| ICU length of stay | 2 (1- 5) | 4 (2-11) | 1.4 | 0.5 to 2.4 | 0.5 | 0.004 |  | 1.3 | 0.5 to 2.0 | 0.4 | 0.001 |
| Duration of IMV in days | 3 (1-5) | 5 (2-11.5) | 1.2 | 0.5 to 2.0 | 0.4 | 0.001 |  | 1.1 | 0.5 to 1.7 | 0.3 | <0.001 |
| Duration of epidural analgesia | 5 (3-6) | 5 (3-8) | -0.3 | -1.0 to 0.4 | 0.3 | 0.432 |  | 0.5 | -0.1 to 1.1 | 0.3 | 0.112 |
| Duration of IV analgesia | 2 (1-5) | 4 (2-6.5) | 0.9 | -0.3 to 2.1 | 0.6 | 0.154 |  | 0.8 | -0.2 to 1.8 | 0.5 | 0.098 |
| NRS (Pain) |  |  |  |  |  |  |  |  |  |  |  |
| Day 3 | 2 (1-3) | 2 (1-3) | -0.4 | -0.9 to 0.1 | 0.3 | 0.139 |  | -0.4 | -0.9 to 0.1 | 0.2 | 0.089 |
| Day 5 | 2 (1-3) | 2 (0-3) | -0.0 | -0.6 to 0.5 | 0.3 | 0.902 |  | 0.1 | -0.4 to 0.5 | 0.2 | 0.721 |
| Day 7 | 2 (1-3) | 2 (0-3) | 0.1 | -0.4 to 0.6 | 0.3 | 0.666 |  | 0.2 | -0.2 to 0.6 | 0.2 | 0.402 |
| In-hospital complications (n, %) |  |  | OR | 95% CI | SE | *p* value |  | OR | 95% CI | SE | *p* value |
| Mortality | 13 (1.5) | 2 (2.5) | 2.3 | 0.1 to 4.7 | 1.5 | 0.575 |  | 2.4 | 0.2 to 29.6 | 1.3 | 0.505 |
| Tracheostomy | 9 (1.1) | 3 (3.8) | 4.0 | 0.3 to 41.2 | 1.2 | 0.247 |  | 2.5 | 0.4 to 16.3 | 0.9 | 0.336 |
| Pneumonia | 113 (13.3) | 24 (30.0) | 2.0 | 0.9 to 4.1 | 0.4 | 0.066 |  | 2.4 | 1.2 to 4.6 | 0.3 | 0.012 |
| Pleural effusion | 18 (2.1) | 4 (5.0) | 6.0 | 0.7 to 39.5 | 0.9 | 0.070 |  | NA | NA | NA | NA |
| Pneumothorax | 18 (2.1) | 6 (7.5) | 13.6 | 1.9 to 127.5 | 1.0 | 0.011 |  | NA | NA | NA | NA |
| Hemothorax | 15 (1.8) | 5 (6.2) | 3.0 | 0.6 to 15.5 | 0.8 | 0.163 |  | NA | NA | NA | NA |
| Other complication | 188 (22.2) | 36 (45) | 2.3 | 1.2 to 4.4 | 0.3 | 0.013 |  | 2.5 | 0.4 to 16.3 | 0.9 | 0.336 |
| Follow up 1 year |  |  |  |  |  |  |  |  |  |  |  |
| EQ5D-5L index value | 0.83 ±0.2 | 0.75 ±0.2 | -0.1 | -0.2 to -0.0 | 0.03 | <0.001 |  | -0.1 | -0.1 to -0.0 | 0.02 | 0.003 |
| EQ5D-5L VAS, mean ±SD | 76 ±19 | 73 ±17 | -4.6 | -10.4 to 1.2 | 2.9 | 0.117 |  | -2.3 | -7.7 to 3.0 | 2.7 | 0.394 |
| MMRC, median (IQR) | 0 (0-0) | 0 (0-1) | 0.2 | -0.0 to 0.5 | 1.9 | 0.055 |  | 0.2 | -0.1 to 0.4 | 0.1 | 0.147 |

RF rib fixation, ICU intensive care unit, IMV invasive mechanical ventilation, IV intravenous, NRS numeric rating scale, ARDS, acute respiratory distress syndrome, IQR interquartile range,

b Regression coefficient between rib fixation and non-operative treatment, CI confidence interval, SE standard deviation, NA no answer (due to small numbers)

**Non-operative versus operative treatment for multiple rib fractures after blunt thoracic trauma: a multicenter prospective cohort study**

European Journal of Trauma and Emergency Surgery

Ruben J. Hoepelman,^1^ MD, Frank. J.P. Beeres,^2^ MD, PD, PhD, FEBS, Reinier B. Beks,^1^ MD, PhD, Arthur A.R. Sweet,^1^ MD, Frank F. Ijpma,^3^ MD, PhD, FEBS, Koen W.W. Lansink^4^, MD, PhD, Bas van Wageningen,^5^ MD, Tjarda N. Tromp,^5^ Bsc, Björn-Christian Link,^2^ MD, PhD, Nicole M. van Veelen,^2^ MD, Jochem. M. Hoogendoorn,^6^ MD, PhD Mirjam B. de Jong,^1^ MD, PhD, Mark. C.P. van Baal,^1^ MD, PhD , Luke P.H Leenen,^1^ MD, PhD, FACS, FEBS, Rolf H.H. Groenwold,^7,8^ MD, PhD, and Roderick M. Houwert,^1^ MD, PhD

1. Department of Trauma Surgery, University Medical Center Utrecht, Utrecht, the Netherlands
2. Department of Orthopedic and Trauma Surgery, Luzerner Kantonsspital, Lucerne, Switzerland
3. Department of Trauma surgery, University Medical Center Groningen, University of Groningen, Groningen, the Netherlands
4. Department of Trauma Surgery, Elisabeth-TweeSteden hospital, Tilburg, The Netherlands.
5. Department of Trauma Surgery, Radboud University Medical Center, Nijmegen, the Netherlands
6. Department of Trauma Surgery, Haaglanden Medical Center, the Hague, the Netherlands
7. Department of Clinical Epidemiology, Leiden University Medical Center, Leiden, the Netherlands
8. Department of Biomedical Data Sciences, Leiden University Medical Center, Leiden, the Netherlands

**Corresponding author**

Roderick M. Houwert, MD, PhD

E-mail address: r.m.houwert@umcutrecht.nl
