# Supplementary material for: Non-operative vs. operative treatment for multiple rib fractures after blunt thoracic trauma: a multicenter prospective cohort study
Source: Eur J Trauma Emerg Surg. 2022 Aug 25;49(1):461–71. doi: 10.1007/s00068-022-02093-9 (PMC9925506; doi:10.1007/s00068-022-02093-9)
Supplement: Supplementary file 7 — Supplementary file7 (DOCX 21 KB) [file 68_2022_2093_MOESM7_ESM.docx]

**Supplementary Table 6** Outcomes before propensity score matching

| Outcome variable | Nonoperative (n=847) | Rib fixation (n=80) |
| --- | --- | --- |
| Hospital length of stay | 7 (4-13) | 12 (8-18) |
| Hospital length of stay from RF | 6 (2-12) | 8 (6-15) |
| Intensive care treatment (n, %) | 228 (26.9) | 27 (33.8) |
| Duration of ICU stay in days | 2 (1- 5) | 4 (2-11) |
| Need for mechanical ventilation (n, %) | 115 (13.6) | 20 (25) |
| Duration of IMV in days | 3 (1-5) | 5 (2-11.5) |
| Tracheotomy (n, %) | 9 (1.1) | 3 (3.8) |
| Epidural analgesia (n, %) | 139 (16.4) | 26 (32.5) |
| Duration of epidural analgesia | 5 (3-6) | 5 (3-8) |
| Duration of IV analgesia | 2 (1-5) | 4 (2-6.5) |
| NRS |  |  |
| Day 3 | 2 (1-3) | 2 (1-3) |
| Day 5 | 2 (1-3) | 2 (0-3) |
| Day 7 | 2 (1-3) | 2 (0-3) |
| In-hospital complications (n, %) |  |  |
| Complications (n, %) |  |  |
| ARDS | 2 (0.2) | 0 (0) |
| Pneumonia | 113 (13.3) | 24 (30.0) |
| Pneumothorax | 18 (2.1) | 6 (7.5) |
| Pleural effusion | 18 (2.1) | 4 (5.0) |
| Hemothorax | 15 (1.8) | 5 (6.2) |
| Empyema | 2 (0.2) | 3 (3.8) |
| Tension pneumothorax | 2 (0.2) | 1 (1.2) |
| Fracture related infection | 0 (0.0) | 0 (0.0) |
| Surgical site infection | 0 (0.0) | 0 (0.0) |
| Other complication (n, %) | 188 (22.2) | 36 (45) |
| Mortality (n, %) | 13 (1.5) | 2 (2.5) |
| Follow up 6 weeks |  |  |
| EQ5D-5L index value, mean ±SD | 0.73 ±0.2 | 0.71 ±0.2 |
| EQ5D-5L VAS, mean ±SD | 68.1 ±19 | 67.8 ±16 |
| MMRC, median (IQR) | 0 (0-1) | 0.5 (0-1) |
| NRS |  |  |
| General | 2 (1-3) | 2 (1-4) |
| Breathing | 0 (0-2) | 1 (0-3) |
| Coughing | 2 (0-4) | 3 (1-5) |
| Complications (n, %) |  |  |
| Pneumonia | 3 (0.4) | 1 (1.2) |
| Pleural effusion | 7 (0.8) | 1 (1.2) |
| Pneumothorax | 3 (0.4) | 0 (0) |
| Hemothorax | 6 (0.7) | 0 (0) |
| Follow up 1 year |  |  |
| EQ5D-5L index value, mean ±SD | 0.83 ±0.2 | 0.75 ±0.2 |
| EQ5D-5L VAS, mean ±SD | 76 ±19 | 73 ±17 |
| MMRC, median (IQR) | 0 (0-0) | 0 (0-1) |
| NRS (pain) |  |  |
| General | 0 (0-2) | 2 (0-4) |
| Breathing | 0 (0-0) | 0 (0-0) |
| Coughing | 0 (0-0) | 0 (0-1) |
| Complications |  |  |
| Implant related irritation (n,%) | 0 (0) | 22 (27.5) |
| Implant removal (n, %) | 0 (0) | 2 (2.5) |
| Symptomatic non-union | 4 (0.5) | 1 (1.2) |
| Deceased (n, %) | 11 (1.3) | 1 (1.2) |
| Return to work (weeks), median (IQR) | 12 (6-18) | 12 (9.5-20) |
| Return to sports (weeks), median (IQR) | 12 (8-24) | 12 (10-19.5) |

RF rib fixation, ICU intensive care unit, IMV invasive mechanical ventilation, IV intravenous, NRS numeric rating scale, ARDS, acute respiratory distress syndrome, IQR interquartile range, CI confidence interval, SE standard deviation, NA no answer (due to small numbers), MMRC modified medical research council dyspnea scale

**Non-operative versus operative treatment for multiple rib fractures after blunt thoracic trauma: a multicenter prospective cohort study**

European Journal of Trauma and Emergency Surgery

Ruben J. Hoepelman,^1^ MD, Frank. J.P. Beeres,^2^ MD, PD, PhD, FEBS, Reinier B. Beks,^1^ MD, PhD, Arthur A.R. Sweet,^1^ MD, Frank F. Ijpma,^3^ MD, PhD, FEBS, Koen W.W. Lansink^4^, MD, PhD, Bas van Wageningen,^5^ MD, Tjarda N. Tromp,^5^ Bsc, Björn-Christian Link,^2^ MD, PhD, Nicole M. van Veelen,^2^ MD, Jochem. M. Hoogendoorn,^6^ MD, PhD Mirjam B. de Jong,^1^ MD, PhD, Mark. C.P. van Baal,^1^ MD, PhD , Luke P.H Leenen,^1^ MD, PhD, FACS, FEBS, Rolf H.H. Groenwold,^7,8^ MD, PhD, and Roderick M. Houwert,^1^ MD, PhD

1. Department of Trauma Surgery, University Medical Center Utrecht, Utrecht, the Netherlands
2. Department of Orthopedic and Trauma Surgery, Luzerner Kantonsspital, Lucerne, Switzerland
3. Department of Trauma surgery, University Medical Center Groningen, University of Groningen, Groningen, the Netherlands
4. Department of Trauma Surgery, Elisabeth-TweeSteden hospital, Tilburg, The Netherlands.
5. Department of Trauma Surgery, Radboud University Medical Center, Nijmegen, the Netherlands
6. Department of Trauma Surgery, Haaglanden Medical Center, the Hague, the Netherlands
7. Department of Clinical Epidemiology, Leiden University Medical Center, Leiden, the Netherlands
8. Department of Biomedical Data Sciences, Leiden University Medical Center, Leiden, the Netherlands

**Corresponding author**

Roderick M. Houwert, MD, PhD

E-mail address: r.m.houwert@umcutrecht.nl
